# Translational trends and specific nutritional interventions for aromatase inhibitor induced bone loss based on a 25 year multi database bibliometric and thematic evolution analysis from 2000 to 2025

**DOI:** 10.3389/fnut.2026.1969024

**Published:** 2026-10-26

**Authors:** Zhenfan Huang, Yuyu Zhang, Qiongyu Wu, Shaoda Meng

**Affiliations:** Department of Breast and Thyroid Surgery, The First People's Hospital of Yunnan Province, Kunming, China

**Keywords:** aromatase inhibitor-induced bone loss, bibliometrics, breast cancer, nutritional interventions, vitamin D

## Abstract

**Background:**

Accelerated bone loss is often observed in postmenopausal women with breast cancer receiving long-term aromatase inhibitor (AI) therapy; particularly, there is a growing need for targeted nutritional interventions.

**Methods:**

This multi-database bibliometric study evaluated 25 years of literature from 2000 to 2025 retrieved from the Web of Science Core Collection and Scopus. Following PRISMA guidelines, 768 publications were assessed using R-based analytical workflows to examine output trends, collaboration networks, thematic evolution, and citation impact.

**Results:**

Annual scientific production expanded between 2005 and reached a peak in 2011, followed by a stable plateau of annual output through 2025. With strong institutional networks in North America and Europe, the United States was the productivity leader. A conceptual transition from isolated antiresorptive medications (bisphosphonates, denosumab) to an integrative bone microenvironment preservation strategy was suggested by keyword and thematic analyses. Keyword patterns suggested that Vitamin D and calcium are frequently integrated as universal baseline co-therapies, and topics including obesity, treatment-associated arthralgia, and survivorship exhibited high citation impact.

**Conclusion:**

Studies on AI-associated bone loss (AIBL) have moved beyond routine BMD measurement to the structured co-management of micronutrients and metabolism, which may in turn guide future breast cancer survivorship research.

## 1 Introduction

Breast cancer is the most frequently diagnosed cancer in women worldwide, and hormone receptor-positive (HR+) disease accounts for approximately 70–80% of all clinical cases (1, 2). Third-generation aromatase inhibitors (anastrozole, letrozole, and exemestane) are the primary adjuvant endocrine therapies in postmenopausal women with early-stage HR + breast cancer, and have been suggested to considerably decrease disease recurrence and improve long-term survival (3). However, these agents also block the peripheral conversion of androgen precursors to estrogens, accompanying a substantial reduction in systemic estrogens (4). Estrogen possesses a physiological role in controlling osteoclast activity and suppressing bone resorption, so long-term use of AIs is frequently linked to accelerated bone turnover and the development of aromatase inhibitor-induced bone loss (AIBL) (5). BMD loss rates with aromatase inhibitors are 2–4 times higher than those seen in natural postmenopause, and fragility fracture risk as well as impairments in health-related quality of life are subsequently observed (6, 7).

Although effective antiresorptive drugs (bisphosphonates and denosumab) are currently recommended for AIBL in clinical guidelines, long-term clinical use of these drugs may be limited by potential serious adverse events, including atypical femoral fractures, osteonecrosis of the jaw, and severe hypocalcemia (8, 9). Nutritional and supportive microenvironment interventions have therefore gained increasing attention in recent years as complementary approaches to preserve bone health in the long term. Elemental calcium and vitamin D are critical micronutrients that serve as physiologic co-therapies for the intestinal transport of calcium and mineralization of bone (10). Additionally, recent studies have highlighted the biological effects of different dietary patterns, bioactive phytoestrogens, and metabolites generated by the gut microbiota on regulating the signaling pathways of osteoclastogenesis and systemic low-grade inflammation (11, 12). It is therefore valuable to clarify the influences that specific nutritional factors may exert on the bone metabolism microenvironment in order to establish management strategies with balanced nutritional support and low toxicity during long-term endocrine therapy (13).

Over the past 25 years (2000–2025), research focusing on AI-associated bone loss and supportive management has expanded significantly, yielding an extensive and fragmented literature body distributed across medical oncology, endocrinology, bone metabolism, and clinical nutrition. Even though there is an increasing number of clinical trials and narrative overviews, existing syntheses remain mostly qualitative, making it challenging to objectively measure systematic research growth, cross-border collaboration, and subtle transitions in themes. Moreover, previous bibliometric studies in oncology have mostly focused on malignant bone metastases or conventional antiresorptive drugs (14, 15), and the translational landscape of targeted micronutritional support and dietary patterns has largely remained unexplored. As a result, the intellectual framework and evolutionary trajectory of nutritional practices in the context of AI-related bone fragility remain poorly systematized across the fields of oncology and nutrition.

The present study addresses this analytical gap through Base R workflows and bibliometric computational modeling, utilizing a multi-database corpus retrieved from the Web of Science Core Collection and Scopus spanning from 2000 to 2025. This study evaluates publication trends over time, international institutional networks, specific thematic paths in nutrition, and direct historical citation networks to construct an evidence-based topological atlas. The goal of these insights is to outline pivotal translational milestones and emerging research domains that can inform evidence-based supportive care strategies and foster interdisciplinary research in the long-term survivorship of breast cancer.

## 2 Materials and methods

### 2.1 Data source and search strategy

A systematic literature search was conducted across multi-database sources, specifically the Web of Science Core Collection (WoSCC, Science Citation Index Expanded) and Scopus, covering the 25-year publication period from January 1, 2000, to December 31, 2025. To capture the full spectrum of translational research on aromatase inhibitor-induced bone loss and specific nutritional management, a comprehensive search query was structured around four logical blocks: (1) aromatase inhibitor therapy (e.g., “aromatase inhibitor*,” “letrozole,” “anastrozole,” “exemestane,” “AIBL”), (2) target malignancy context (“breast cancer,” “breast neoplasm*,” “breast tumor*”), (3) bone metabolism and microenvironment outcomes (“bone loss,” “osteoporosis,” “bone mineral density”, “BMD”, “fracture*,” “osteoclast*,” “osteoblast*,” “bone microenvironment”), and (4) specific nutritional interventions and dietary patterns (“nutrition*,” “diet*,” “dietary pattern*,” “vitamin D,” “cholecalciferol,” “calcium,” “phytoestrogen*,” “isoflavone*,” “genistein,” “daidzein,” “gut microbiota,” “probiotic*,” “polyphenol*,” “supplement*,” “functional food*”). The search was restricted to original research articles and review articles published in the English language.

### 2.2 Literature selection and data harmonization

We retrieved complete bibliographic records (including cited reference lists) from the Web of Science Core Collection in plain text format and from Scopus in BibTeX format; then, in accordance with the PRISMA 2020 statement (Supplementary Table S4) (16), we imported them into the R environment. A custom Base R harmonization protocol was developed to address cross-database schema heterogeneity and prevent automated truncation of fields during multi-database integration, by computing the column union across databases, assigning NA to missing fields, and casting total citations (TC) and publication years (PY) into numeric formats. Further data cleaning steps included sorting the merged dataset hierarchically by citation frequency in descending order, then sequentially deduplicating records based on Digital Object Identifiers (DOIs) and normalized title strings, to remove redundancies across databases while ensuring that the most comprehensive citation records were retained.

### 2.3 Natural language processing and field standardization

To ensure semantic consistency across keyword, thematic, and trend analyses, author keyword fields (DE) underwent rigorous natural language processing (NLP) and standardization. A customized cleaning dictionary was applied using regular expressions to consolidate medical synonyms, singular/plural variations, and abbreviations. For example, “CHOLECALCIFEROL” and “VITAMIN-D” were normalized to “VITAMIN D,” “ISOFLAVONES” to “PHYTOESTROGENS,” “BONE MINERAL DENSITY” to “BMD,” “ZOLEDRONIC ACID” to “BISPHOSPHONATES,” and “AROMATASE INHIBITOR-INDUCED BONE LOSS” to “AIBL.” Furthermore, database-generated indexing terms, non-specific demographic descriptors (such as “HUMANS,” “FEMALE,” “MIDDLE AGED”), and publication metadata tags (such as “ARTICLE,” “REVIEW,” “PRIORITY JOURNAL”) were systematically removed to eliminate indexing noise. The complete customized R script detailing the data harmonization algorithms, deduplication parameters, and comprehensive NLP cleaning dictionaries is provided in Supplementary File 1 to ensure full methodological reproducibility.

### 2.4 Statistical analysis

Data were analyzed, metrics extracted, and visualizations generated using R software (version 4.3.2). The bibliometrix, igraph, ggrepel, treemapify, and ggplot2 packages were utilized. Custom Base R algorithms were used to calculate basic descriptive bibliometric parameters, such as the total number of publications (TP), the total number of citations (TC), the average number of citations per document, and the author-level Hirsch index (H-index). Notably, Local Citation Scores (LCS) were computed exclusively using the WoS dataset to prevent cross-database reference formatting incompatibilities, whereas Global Citation Scores (GCS) utilized the merged dataset. The sources of publications were classified into core, expansion, and peripheral zones based on Bradford’s Law, which estimates the exponentially diminishing returns of extending a search for references in scientific journals (17). Adjacency matrices were converted to undirected igraph objects for topological network analyses (co-authorship, institutional collaboration, and keyword co-occurrence). To mitigate the influence of isolated nodes and network crowding, component analysis was applied to extract giant connected components. Spatial layouts of networks were derived using the Fruchterman-Reingold force-directed algorithm, and text label collisions were minimized by automated repulsion algorithms. Strategic thematic maps were created based on the assessment of centrality (relevance degree) and density (development degree) at the keyword level.

## 3 Results

### 3.1 Primary literature yield and journal production dynamics

The initial number of raw records retrieved was 934 from the Web of Science Core Collection and Scopus databases was 1,220. During the title, abstract, and thematic eligibility screening phase, 286 records were excluded (identifying off-target topics, non-breast cancer studies, and ineligible publication types). Following the retrieval of the remaining 934 eligible records for database integration, 166 duplicate articles were removed. Ultimately, a finalized dataset of 768 original articles and reviews between 2000 and 2025 was included for systematic analysis (Figure 1A). This literature body originated from 366 academic journals and was written by 3,558 investigators who, on average, received 44.89 citations per document and cited 5,719 unique references (Supplementary Table S1).

**Figure 1:**
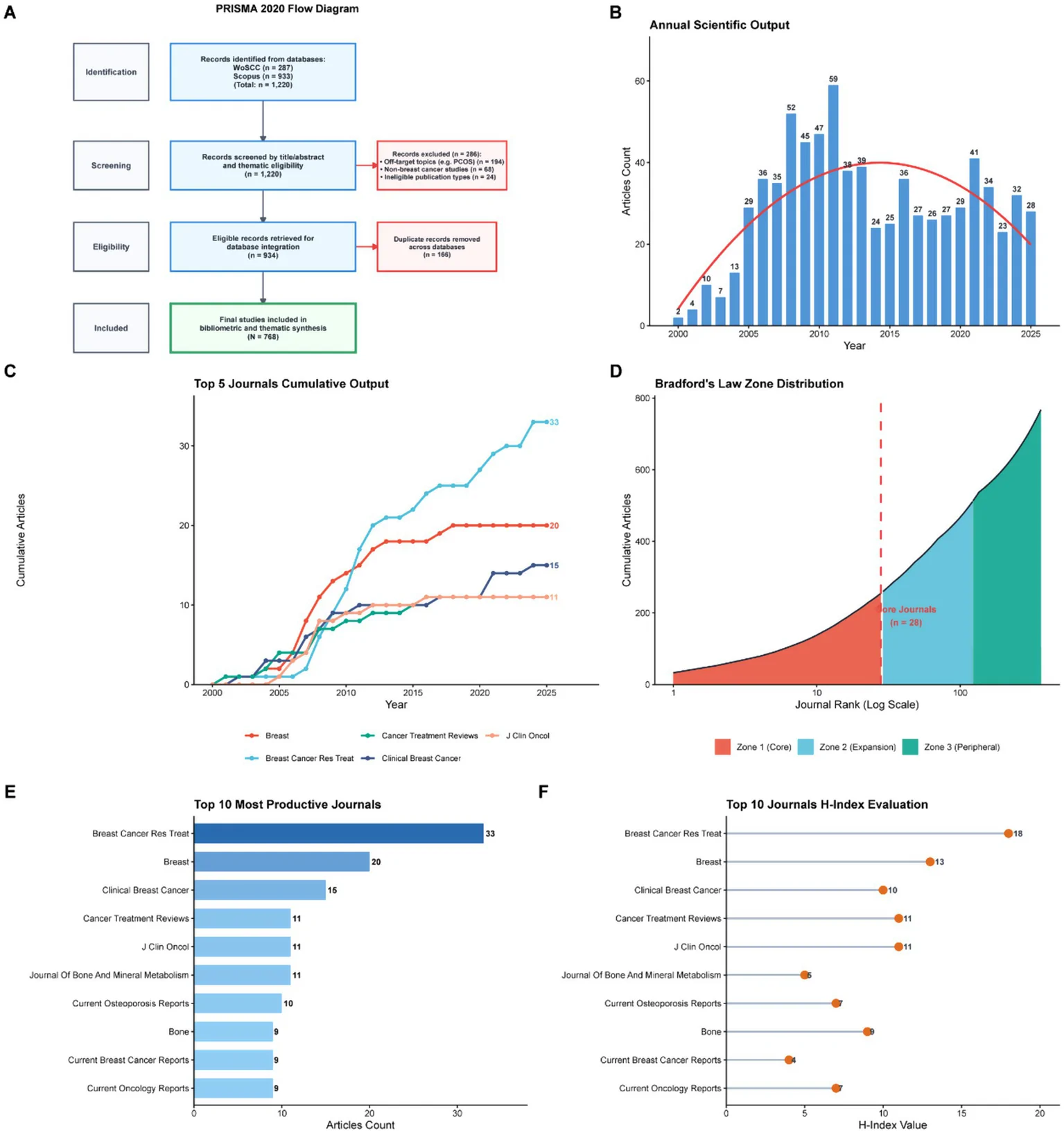
Overview of literature selection and dynamics of core journal output. **(A)** PRISMA 2020 flow diagram detailing record identification, eligibility screening, duplicate removal, and final study selection (*N* = 768). **(B)** Scientific production for the years 2000 to 2025 with a polynomial trend line. **(C)** Cumulative publication dynamics of the top 5 core journals over time. **(D)** Bradford’s Law zone distribution of core, expansion, and peripheral journal partitions. **(E)** Top 10 most productive journals according to the number of articles. **(F)** H-index evaluation of the top 10 productive journals.

The annual scientific production showed a progressive upward trend during the 25-year observation period (Figure 1B). Publication volume increased from 2 articles in 2000, subsequently entering a growth phase from 2005 to 2011, with 2011 recording a peak of 59 articles. Subsequently, there was a period of stable annual output, ranging from 23 to 41 articles per year through 2025 (Figure 1B).

Bradford’s Law of literature dispersion indicated that there were 28 core journals (Zone 1) that published about one-third of the literature (Figure 1D). The top 5 core journals exhibited distinct cumulative publication dynamics over time (Figure 1C). Cumulative output displayed a steady upward trend, with Breast Cancer Research and Treatment leading with 33 publications by 2025, followed by Breast (*n* = 20), Clinical Breast Cancer (*n* = 15), Cancer Treatment Reviews (*n* = 11), and Journal of Clinical Oncology (*n* = 11) (Figure 1C). The distribution across 366 different sources suggests that research on bone loss during AI treatment and nutritional interventions is interdisciplinary in nature, encompassing medical oncology, bone metabolism, and supportive clinical nutrition.

The main dissemination venues in this domain were further evaluated through analysis of publication volume and local academic impact (Figures 1E,F). Breast Cancer Research and Treatment ranked highest in terms of total articles published (*n* = 33) and local H-index (H = 18) (Figures 1E,F). Specialized breast disease journals (Breast, *n* = 20, H = 13; Clinical Breast Cancer, *n* = 15, H = 10), in addition to general high-impact oncology journals (Journal of Clinical Oncology, *n* = 11, H = 11; Cancer Treatment Reviews, *n* = 11, H = 11), provided significant scholarly support. Additionally, bone- and metabolic-focused journals, such as Journal of Bone and Mineral Metabolism (*n* = 11, H = 5) and Current Osteoporosis Reports (*n* = 10, H = 7), underscored the integrated aspect of bone health research in supportive cancer care (Figures 1E,F).

### 3.2 Geographical distribution and multi-level collaboration networks

The geographical, institutional, and authorial collaboration networks were systematically analyzed to describe the scientific capacity in this field at the macro level (Figures 2A–F; Supplementary Table S2). In terms of overall publication output, the USA had the largest number (*n* = 303), followed by the United Kingdom (*n* = 84), Italy (*n* = 66), Germany (*n* = 42), Canada (*n* = 36), and Australia (*n* = 34) across all 25 years of publications (Supplementary Table S2). The country collaboration network was a dense network with mainly North American and European countries as nodes, where the USA, United Kingdom, Germany, France, and Italy were the most important topological hubs (Figure 2A). A corresponding author analysis identified different country collaboration patterns (Figure 2B). Of the single country publications (SCP), the USA had the highest absolute number (SCP = 243); however, notable absolute counts of multiple country publications (MCP) were observed in European countries such as Italy (MCP = 11), as well as in Canada (MCP = 12), which represented higher MCP ratios relative to their respective total national outputs (Figure 2B). This is likely related to the numerous domestic clinical trial cooperative groups in the USA, while research work in Europe often requires international clinical trial cooperation in multi-center trials.

**Figure 2:**
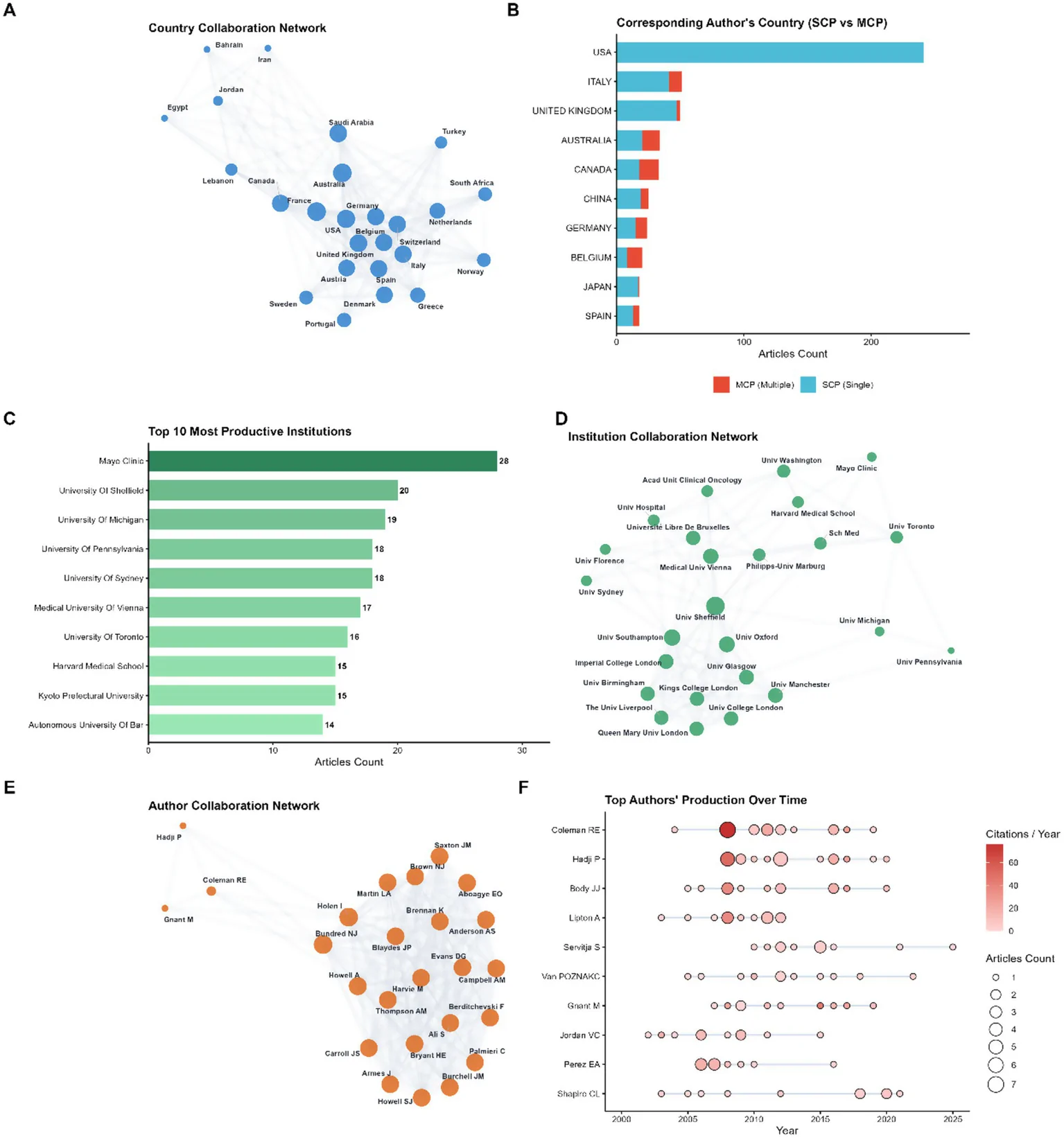
Geographical, institutional, and authorial networks of collaboration. **(A)** Country collaboration network representing geographical co-authorship linkages. **(B)** Country distribution of corresponding author’s single country publications (SCP) and multiple country publications (MCP). **(C)** Top 10 most productive academic institutions (based on number of articles). **(D)** Network of institutions showing co-authorship relationships between academic institutions. **(E)** Author collaboration network with research clusters of key investigators. **(F)** Top authors’ publication timeline from 2000 to 2025, with bubble size indicating annual article counts and color intensity reflecting mean citations per year.

Research productivity at the institutional level was focused in large academic medical centers and groups of affiliated university hospitals (Figures 2C,D; Supplementary Table S2). The institutions with the greatest number of papers were Mayo Clinic (*n* = 28), the University of Sheffield (*n* = 20), the University of Michigan (*n* = 19), the University of Pennsylvania (*n* = 18), and the University of Sydney (*n* = 18) (Figure 2C; Supplementary Table S2). The institutional collaboration network identified distinct transatlantic academic clusters with strong patterns of co-authorship between European institutions (for example the University of Sheffield, Medical University of Vienna) and North American institutions (for example the Mayo Clinic, Harvard Medical School, and University of Toronto) (Figure 2D). Such institutional connections suggest that collaborative academic networks have been an essential part of large-scale prospective assessments of bone mineral loss and supportive bone-targeted regimens.

Key scientific investigators were further identified based on author productivity and local academic impact (Figures 2E,F; Supplementary Table S2). Robert E. Coleman (*n* = 20, TC = 2,643, H = 16) and Peyton Hadji (*n* = 20, TC = 2,156, H = 16) led in publication volume and total citation impact, alongside prominent contributions from Jean-Jacques Body (*n* = 13, TC = 1,713, H = 12), Michael Gnant (*n* = 10, TC = 1,726, H = 8), and V. Craig Jordan (*n* = 10, TC = 1,035, H = 8) (Supplementary Table S2). The author collaboration network showed well-defined clusters of research areas (Figure 2E). Of interest, clinical investigators Hadji P, Coleman RE, and Gnant M clustered tightly, and were joint leaders on international clinical expert panels and key bone-targeted intervention trials. By contrast, other basic research scientists like Jordan VC were depicted as separate nodes, which reflects their interest in the basic pharmacology of estrogen receptors and not clinical trial management. Longitudinal analysis showed that authors with a history of publications had a stable number of publications and citations per year throughout the mid-2000s to recent years, while investigators Servitja S and Van Poznak C demonstrated publication activity in recent years (Figure 2F).

### 3.3 Keyword co-occurrence structure, thematic evolution, and citation impact

Author keyword distributions and co-occurrence patterns were systematically assessed to understand the intellectual structure, specific nutritional interventions, and thematic evolution (Figures 3A–F). The most frequent terms found with the keyword treemap were Osteoporosis (*n* = 139), Bisphosphonates (*n* = 117), Denosumab (*n* = 64), BMD (*n* = 62), Tamoxifen (*n* = 54), Bone Loss (*n* = 36), Letrozole (*n* = 35), and Vitamin D (*n* = 31) (Figure 3A). The keyword co-occurrence network showed that the network was cohesive with three interconnected conceptual domains: pharmacological bone-targeted agents (Bisphosphonates, Denosumab), endocrine therapeutic modalities (Tamoxifen, Letrozole, Anastrozole, Exemestane), and supportive/nutritional management (Vitamin D, Calcium, Nutrition, Exercise, Quality Of Life, Arthralgia) (Figure 3B). As indicated by this co-occurrence structure, micronutritional interventions are usually explored along with bone mineral density monitoring and antiresorptive pharmacological agents.

**Figure 3:**
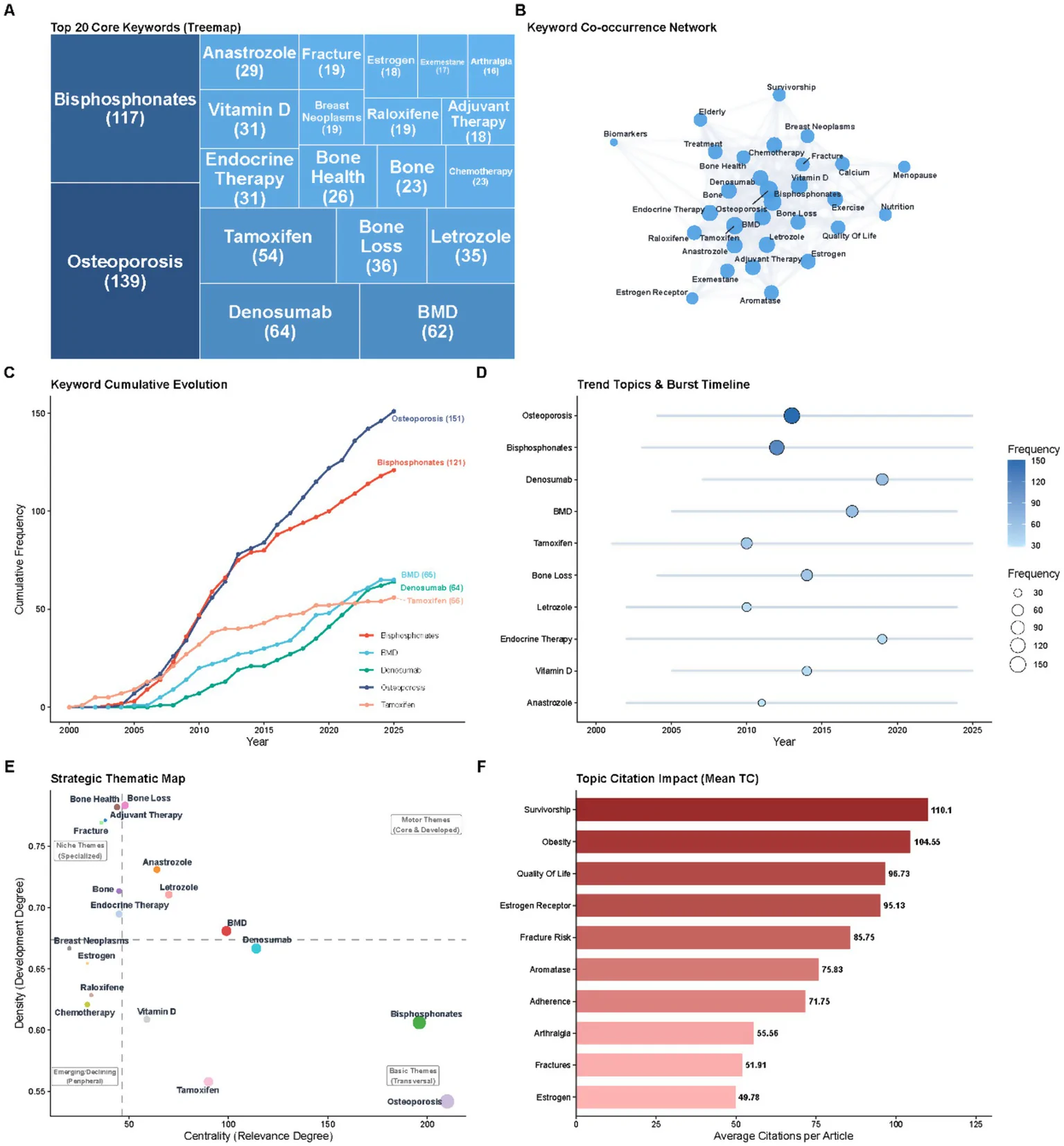
Co-occurrence of keywords, thematic evolution, and citation impact. **(A)** Top 20 core keywords within a treemap showing the relative frequency with which the keywords appear. **(B)** Keyword co-occurrence network showing conceptual connections between author keywords. **(C)** Cumulative publication evolution of top 5 keywords (2000–2025). **(D)** Trend topics & burst timeline showing the median year of publication and active time periods of keywords. **(E)** Strategic thematic map with four quadrants: motor, niche, emerging/declining, and basic themes, on the basis of centrality and density. **(F)** Citation impact with respect to average citations per article (Mean TC) of key words in the topic, where Mean TC represents the total citations received divided by the number of articles containing the specific keyword, reflecting average academic influence.

Over the years (2000–2025), the research focus has evolved over time as shown by the longitudinal mapping of core keywords (Figures 3C,D). Cumulative publication curves revealed that Osteoporosis and Bisphosphonates had an early growth pattern with totals of 151 and 121 occurrences by 2025, respectively (Figure 3C). Denosumab and BMD, on the other hand, showed significant acceleration after 2010, with each having a cumulative count of 64 and 65, respectively (Figure 3C). The evolution was further illustrated by the trend topics timeline (Figure 3D) and visually mapped via an alluvial diagram across three distinct translational eras (Supplementary Figure S1). Early studies were mainly about Tamoxifen (a selective estrogen receptor modulator, median year of publication ~2010) and first-generation bisphosphonates, while later periods of activity were dedicated to RANK-L inhibition (Denosumab, median year of publication ~2019), stringent monitoring of BMD (median year of publication ~2017), and supplemental Vitamin D (median year of publication ~2014) (Figure 3D). The patterns indicate the shift from general antiresorptive treatment towards targeted biologics and well-defined bone microenvironment preservation.

To strengthen the focus on targeted dietary support, a sub-analysis restricted to nutrition-specific keywords was conducted (Supplementary Table S5). While broad pharmacological terms dominated the general network, the isolated nutritional landscape revealed that Vitamin D (*n* = 31) and Calcium (*n* = 28) constituted the fundamental micronutritional axis. Notably, emerging dietary and metabolic regulators demonstrated growing relevance, including Phytoestrogens and Isoflavones (*n* = 14), Gut Microbiota and Probiotics (*n* = 11), and Polyphenols (*n* = 9). The co-occurrence patterns within this sub-network suggest that contemporary research is gradually expanding from basic elemental supplementation toward modulating the gut-bone axis and exploring the osteoprotective potential of plant-derived bioactive compounds in mitigating treatment-induced inflammation.

All core topics have been strategically mapped by taking into account Callon’s centrality (relevance degree) and density (development degree) (Figure 3E). Osteoporosis and Bisphosphonates lay in the Basic Themes quadrant (high centrality, low density), indicating that they are pillars of the field that cross the entire field (Figure 3E). BMD, Denosumab, and Letrozole occupied the Motor Themes quadrant (high centrality, high density), and these entities were also well-developed drivers of contemporary research. The specialized clinical concepts like Bone Loss, Bone Health, and Adjuvant Therapy were positioned as Niche Themes. In particular, baseline nutritional factors (Vitamin D) and endocrine agents (Tamoxifen and Estrogen) were in the lower-left quadrant (Figure 3E). The placement of Vitamin D in this quadrant suggests a lower relative network novelty compared to actively researched pharmaceutical agents; however, it remains a frequently co-occurring term alongside foundational clinical concepts, which may reflect its established role rather than a decline in overall scientific relevance.

Assessment of topic citation impact (Mean TC) brought to light important intellectual concepts that are supporting field recognition (Figure 3F). The highest citation impacts were achieved by Survivorship (Mean TC = 110.10), Obesity (Mean TC = 104.55), Quality Of Life (Mean TC = 96.73), Estrogen Receptor (Mean TC = 95.13), Fracture Risk (Mean TC = 85.75), and Arthralgia (Mean TC = 55.56) (Figure 3F). Obesity, Arthralgia, and Survivorship’s high citation impact reflects the clinical interconnections between peripheral metabolic status, musculoskeletal treatment-associated toxicities, and adherence to treatment.

### 3.4 Citation lineage, co-citation infrastructure, and local versus global impact

The historical direct citation paths and co-citation structures were systematically analyzed to understand the chronological development of the research field and reveal the intellectual landscape underpinning it (Figures 4A–F; Supplementary Table S3). A well-defined historical sequence was identified from initial pioneering safety trials to organized clinical and lifestyle management programs (Figure 4A). Primary clinical recognition of aromatase inhibitor (AI) associated bone loss was established by early foundational publications that included Chlebowski (41) and Perez (18). These works directly informed subsequent multi-center trial evaluations that have been conducted (29) Van Poznak (19), and ultimately supported integrative lifestyle, metabolic, and supportive intervention protocols (30, 40) (Figure 4A). There is a logical progression from initial risk identification to comprehensive supportive bone preservation strategies as a result of this direct citation lineage.

**Figure 4:**
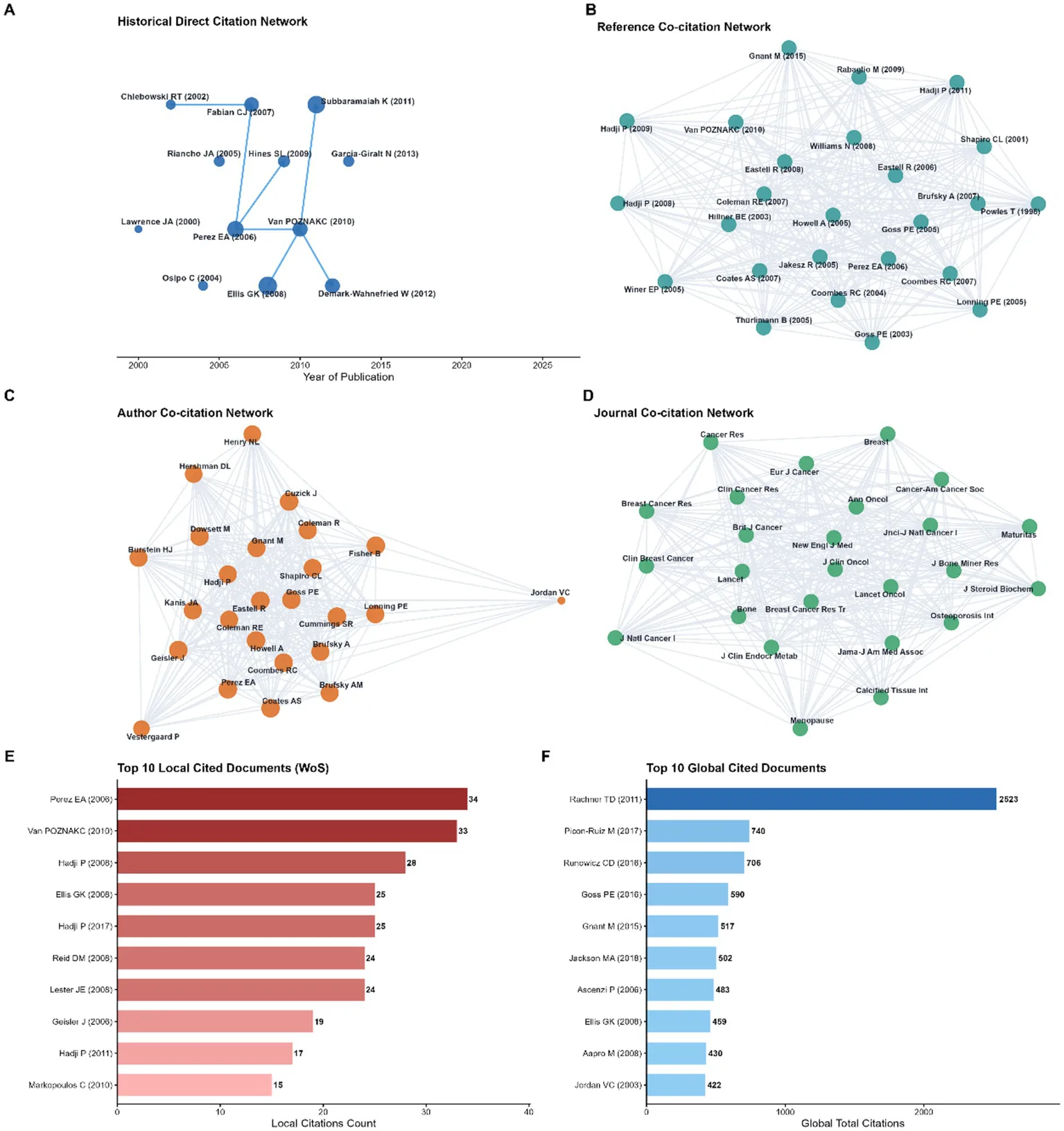
Historical direct citing structure and co-citation infrastructure. **(A)** Historical direct citation network of chronological direct reference flows between key publications. **(B)** Co-citation network showing underlying literature co-cited by included studies. Co-citation network of authors **(C)** that frequently co-cite to each other. **(D)** Co-citation relationship of the primary publishing sources presented in Journal co-citation network. **(E)** Top 10 local cited documents ranked by number of citations in the current Web of Science collection (Local Citations). **(F)** Most cited documents in the world by all citations (Global Citations).

Co-citation network analysis was also used to uncover the underlying intellectual infrastructure (Figures 4B–D). The reference co-citation network revealed landmark clinical trial reports [mostly Gnant (31), Hadji (32), Coleman (33), and Goss (34)] as core co-cited nodes that are often cited together to form consensus management algorithms (Figure 4B). The author co-citation network revealed frequently co-cited investigators such as Hadji P, Coleman RE, Eastell R, Goss PE, and Jordan VC, who were all influential in the development of clinical guidelines and endocrine safety practices (Figure 4C). In addition, the journal co-citation network showed that there was a dual-pillar “intellectual bridge” between the key clinical oncology journals (Journal of Clinical Oncology, The New England Journal of Medicine, The Lancet, Annals of Oncology) and the specialized bone metabolism journals (Bone, Osteoporosis International, Journal of Bone and Mineral Research) (Figure 4D).

Comparative evaluation of local citation frequency (LCS, within the Web of Science dataset exclusively) and global citation frequency (GCS, across the merged indexed literature) showed different aspects of scholarly impact, though comparisons should be interpreted with the caveat that LCS reflects an intra-database metric while GCS reflects broader multi-database reach (Figures 4E,F; Supplementary Table S3). Perez (18) (LCS = 34) and Van Poznak (19) (LCS = 33) recorded the highest local citation counts, followed by Hadji (35) (LCS = 28), Ellis (2008) (LCS = 25), and Hadji (36) (LCS = 25) (Figure 4E). Publications with high local citation values suggest that they were used as clinical benchmarks in the specialized literature and were cited by subsequent studies in this defined group of literature. By comparison, citations in the global ranking were dominated by more general biological/survivorship publications such as Rachner (20) (The Lancet, GCS = 2,523), Picon-Ruiz (21) (CA: A Cancer Journal for Clinicians, GCS = 740), Runowicz (37) (GCS = 706), Goss (38) (GCS = 590), Gnant (31) (GCS = 517), and Jackson (39) (Nature Communications, GCS = 502) (Figure 4F; Supplementary Table S3). The difference between LCS and GCS metrics indicates that globally published literature offers general mechanistic, metabolic, and survivorship frameworks, while locally published literature serves as specific operational guidelines directly relevant to clinical management of aromatase inhibitor- induced bone loss and nutritional co-therapies.

## 4 Discussion

Although third-generation aromatase inhibitors (AI) are the current standard of adjuvant endocrine therapy for postmenopausal women with hormone receptor-positive breast cancer, therapy-induced estrogen deprivation is often associated with elevated risks of bone loss and fractures, which requires dedicated supportive management (22, 23). This 25-year multi-database bibliometric study traced the evolution of research on AI-associated bone loss and the development of specific nutrition-based strategies for microenvironment preservation, to meet the clinical challenge. The analysis reveals how the field evolved from the simple monitoring of bone density towards the development of well-defined management guidelines that involve micronutrients, regulators of metabolism, and specific antiresorptive medicines.

The temporal growth pattern and the publication landscape by country mirrors the evolution of bone safety measures in the clinical setting of oncology. The first output increase seen between 2005 and 2011 seems to coincide with the main publication of pivotal randomized controlled trials demonstrating the efficacy of aromatase inhibitors (ATAC, BIG 1–98), which were associated with increased awareness of the effects of treatment on bone fragility (24, 25). The stable output phase from 2012 to 2025 indicates that basic management principles are accepted as a clinical consensus and research has now turned to more specialist nutritional and metabolic co-therapies, suggesting a transition from initial epidemiological characterization to more refined mechanistic investigation and specific nutritional management strategies. Geographically, the United States had the highest number of publications and single country publications, which aligns with bibliometric analysis in the broader supportive cancer care. This strong domestic productivity is probably a result of the centralized infrastructure of national cooperative clinical trial groups funded by the Federal government. By contrast, European countries had more multiple-country publications, which is likely to be due to the strong collaboration across countries in the region, particularly through the European Organisation for Research and Treatment of Cancer and the Austrian Breast and Colorectal Cancer Study Group.

The co-occurrence of key words and strategic thematic coordinates indicated that there was a shift from a single antiresorptive therapy to the maintenance of the bone microenvironment. Although initial studies mainly addressed bisphosphonates, more recent publications highlight the use of specific biological drugs (e.g., denosumab) along with background nutritional care (26). Interestingly, Vitamin D was placed in the bottom left corner of the strategic thematic map which may seem a little strange for a critical supporting intervention. But when compared to clinical management guidelines, vitamin D and calcium supplementation have been universally applied as a baseline protocol in almost all bone clinical trials, so it is not a lack of clinical utility, but rather lower relative network centrality, compared to actively tested prescription pharmaceuticals (27). Moreover, there was a high impact of topics like obesity and arthralgia, reflecting the increasing awareness of the systemic metabolic status and treatment toxicity. Since musculoskeletal symptoms associated with aromatase inhibitors (AIs) are often cited as a major reason for poor adherence to treatment, managing peripheral estrogen conversion as well as joint toxicity may be directly related to survivorship (28), indicating that musculoskeletal symptoms and metabolic bone health are emerging as important factors in long-term breast cancer survivorship.

Local citation frequency and global total citations reflect two complementary aspects of academic influence. Domain specific clinical guidelines and consensus papers, such as the reports by Perez et al. and Van Poznak et al., were identified as key operational benchmarks, as cited directly in this specialized literature cohort, through local citation metrics (18, 19). In contrast, the most cited articles worldwide were broad narrative reviews and general clinical guidelines (e.g., Rachner et al. on mechanisms of osteoporosis and Picon-Ruiz et al. on obesity), and have a cross-disciplinary impact on both endocrinology and oncology (20, 21). This structural variation implies that the literature that is cited globally provides a general understanding of the pathophysiological background, while literature cited locally takes the form of instructions for action, or operational protocols, used in everyday clinical decision-making on bone health in the context of endocrine therapy.

Several key limitations of this study must be taken into account when interpreting the results. First, only English language publications in the Web of Science Core Collection and Scopus were retrieved from literature, so the most recent cutting-edge clinical trials or new findings on the gut microbiota-bone axis could have lower absolute citation numbers. Third, while rigorous data harmonization was applied, the reliance on metadata without manual full-text qualitative screening means that bibliometric analyses cannot assess the methodological quality or clinical efficacy of the included studies. Fourth, keyword co-occurrence analysis inherently captures dominant terms; thus, concepts buried within titles or abstracts may be underrepresented, and the relatively low frequency of highly specific dietary terms limits the ability to draw robust conclusions regarding specific food-based patterns. Finally, the observed productivity patterns exhibit a geographical bias toward Western countries, which may underrepresent research perspectives and genetic diversities from other global regions.

## 5 Conclusion

The present 25-year bibliometric study offers a broad perspective on the trends of research on bone loss associated with aromatase inhibitors and nutritional interventions designed to support bone health, from 2000 to 2025. The results suggest a transition from early bone mineral density assessment to the use of single antiresorptive drugs to integrative approaches that also include micronutritional co-therapies, specific biologics, and systemic modulation of the metabolic environment. There remains a focus on North America and Europe for international research and a two-pronged intellectual base: medical oncology and bone metabolism. Future initiatives should aim to foster broader participation and infrastructure development in developing regions across Asia, Africa, and Latin America, which is essential for capturing a truly global epidemiological trend and addressing diverse population-specific nutritional needs. Whereas core interventions like vitamin D, calcium, bisphosphonates, and denosumab have become accepted in clinical practice, thematic trends indicate a rising interest in systemic metabolic factors, including obesity and treatment-related toxicity. Future studies may be enhanced by exploring tailored nutrition plans, such as the gut microbiota-bone axis, in order to optimize bone microenvironment preservation and to promote long-term survivorship in hormone receptor-positive breast cancer care.

## Data Availability

The original contributions presented in the study are included in the article/Supplementary material, further inquiries can be directed to the corresponding author.
