# Supplementary material for: Translational trends and specific nutritional interventions for aromatase inhibitor induced bone loss based on a 25 year multi database bibliometric and thematic evolution analysis from 2000 to 2025
Source: Front Nutr. 2026 Oct 26;13:1969024. doi: 10.3389/fnut.2026.1969024 (PMC13745266; doi:10.3389/fnut.2026.1969024)
Supplement: Supplementary file 3 [file Supplementary_file_1.DOCX]

**Supplementary Figure S1. Alluvial diagram tracking the thematic evolution of core concepts across translational eras.**

This visualization illustrates the dynamic flow and frequency shifts of the most prominent author keywords spanning three developmental phases in aromatase inhibitor-associated bone loss research: Early Exploration (2000–2010), Pharmacological Era (2011–2018), and Integrative Care (2019–2025). The width of each horizontal flow segment (alluvium) corresponds to the keyword's relative publication frequency during that specific period. The diagram highlights the historical transition from conventional antiresorptive dependence (e.g., Bisphosphonates) toward the prominent adoption of targeted biologics (e.g., Denosumab), while demonstrating the sustained integration of foundational support therapies (e.g., Vitamin D). (Note: "Prostate Cancer" appears as a parallel clinical cohort frequently co-investigated alongside breast cancer regarding treatment-induced bone loss).
