# Supplementary material for: Translational trends and specific nutritional interventions for aromatase inhibitor induced bone loss based on a 25 year multi database bibliometric and thematic evolution analysis from 2000 to 2025
Source: Front Nutr. 2026 Oct 26;13:1969024. doi: 10.3389/fnut.2026.1969024 (PMC13745266; doi:10.3389/fnut.2026.1969024)
Supplement: Supplementary file 4 [file Supplementary_file_2.DOCX]

Table S1. Main information regarding research output on AIBL and nutritional interventions (2000–2025).

| **Metric** | **Value** |
| --- | --- |
| **Timespan** | 2000 – 2025 |
| **Total Documents** | 768 |
| **Sources (Journals)** | 366 |
| **Total Authors** | 3,558 |
| **Average Citations per Document** | 44.89 |
| **Total Cited References** | 5,719 |

Note: Data extracted from Web of Science Core Collection and Scopus databases after deduplication and quality filtering.

Table S2. Top 10 most productive authors, affiliations, and countries/regions.

| **Rank** | **Author** | **Author TP** | **Author TC** | **Author *h*-index** | **Affiliation** | **Affiliation TP** | **Country/Region** | **Country TP** |
| --- | --- | --- | --- | --- | --- | --- | --- | --- |
| **1** | Coleman RE | 20 | 2,643 | 16 | Mayo Clinic | 28 | USA | 303 |
| **2** | Hadji P | 20 | 2,156 | 16 | University of Sheffield | 20 | United Kingdom | 84 |
| **3** | Body JJ | 13 | 1,713 | 12 | University of Michigan | 19 | Italy | 66 |
| **4** | Lipton A | 13 | 1,226 | 11 | University of Pennsylvania | 18 | Germany | 42 |
| **5** | Van Poznak C | 11 | 375 | 8 | University of Sydney | 18 | Canada | 36 |
| **6** | Servitja S | 11 | 324 | 8 | Medical University of Vienna | 17 | Australia | 34 |
| **7** | Gnant M | 10 | 1,726 | 8 | University of Toronto | 16 | Belgium | 28 |
| **8** | Jordan VC | 10 | 1,035 | 8 | Harvard Medical School | 15 | Spain | 28 |
| **9** | Perez EA | 10 | 1,008 | 9 | Kyoto Prefectural University of Medicine | 15 | China | 25 |
| **10** | Shapiro CL | 10 | 385 | 8 | Autonomous University of Barcelona | 14 | Switzerland | 25 |

Abbreviations: TP, Total Publications; TC, Total Citations; h-index, Hirsch index calculated specifically within this dataset.

Note: Country TP is calculated on an article-level basis (count per document = 1).

Table S3. Top 10 highly cited documents in translational research on AIBL and bone microenvironment.

| **Rank** | **Document Title** | **First Author** | **Journal** | **Year** | **DOI** | **Total Citations** | **Annual Citations** |
| --- | --- | --- | --- | --- | --- | --- | --- |
| **1** | Osteoporosis: Now and the Future | Rachner TD | *The Lancet* | 2011 | 10.1016/S0140-6736(10)62349-5 | 2,523 | 157.69 |
| **2** | Obesity and Adverse Breast Cancer Risk and Outcome: Mechanistic Insights and Strategies for Intervention | Picon-Ruiz M | *CA: A Cancer Journal for Clinicians* | 2017 | 10.3322/caac.21405 | 740 | 74.00 |
| **3** | American Cancer Society/American Society of Clinical Oncology Breast Cancer Survivorship Care Guideline | Runowicz CD | *CA: A Cancer Journal for Clinicians* | 2016 | 10.3322/caac.21319 | 706 | 64.18 |
| **4** | Extending Aromatase-Inhibitor Adjuvant Therapy to 10 Years | Goss PE | *The New England Journal of Medicine* | 2016 | 10.1056/NEJMoa1604700 | 590 | 53.64 |
| **5** | Adjuvant Denosumab in Breast Cancer (ABCSG-18): A Multicentre, Randomised, Double-Blind, Placebo-Controlled Trial | Gnant M | *The Lancet* | 2015 | 10.1016/S0140-6736(15)60995-3 | 517 | 43.08 |
| **6** | Gut Microbiota Associations with Common Diseases and Prescription Medications in a Population-Based Cohort | Jackson MA | *Nature Communications* | 2018 | 10.1038/s41467-018-05184-7 | 502 | 55.78 |
| **7** | Structure-Function Relationship of Estrogen Receptor  $\alpha$   and  $\beta$  : Impact on Human Health | Ascenzi P | *Molecular Aspects of Medicine* | 2006 | 10.1016/j.mam.2006.07.001 | 483 | 23.00 |
| **8** | Randomized Trial of Denosumab in Patients Receiving Adjuvant Aromatase Inhibitors for Nonmetastatic Breast Cancer | Ellis GK | *Journal of Clinical Oncology* | 2008 | 10.1200/JCO.2008.16.3832 | 459 | 24.16 |
| **9** | Guidance on the Use of Bisphosphonates in Solid Tumours: Recommendations of an International Expert Panel | Aapro M | *Annals of Oncology* | 2008 | 10.1093/annonc/mdm442 | 430 | 22.63 |
| **10** | Antiestrogens and Selective Estrogen Receptor Modulators as Multifunctional Medicines. 2. Clinical Considerations and New Agents | Jordan VC | *Journal of Medicinal Chemistry* | 2003 | 10.1021/jm020450x | 422 | 17.58 |

Abbreviations: DOI, Digital Object Identifier.

Note: Annual Citations = Total Citations / (2026 - Publication Year + 1).

Table S5. Sub-analysis of specific nutritional, dietary, and microbiota-related interventions and keywords in AIBL research (2000–2025).

| **Category / Domain** | **Specific Nutrient / Keyword Entity** | **Frequency (*n*)** | **Corpus Proportion (%)** | **Primary Proposed Biological / Bone Mechanism** | **Top Co-occurring Clinical Terms** | **Representative Benchmark References** |
| --- | --- | --- | --- | --- | --- | --- |
| **Essential Micronutrients** | **Vitamin D** *(Cholecalciferol, Ergocalciferol)* | 31 | 4.04% | Promotes intestinal calcium absorption via VDR; suppresses PTH secretion; attenuates osteoclastic resorption | BMD, Osteoporosis, Calcium, Bisphosphonates | Fleet (2022); Hadji et al. (2025) |
|  | **Calcium** *(Elemental calcium, Carbonate, Citrate)* | 28 | 3.65% | Substrate for hydroxyapatite crystal formation; preserves bone mineral matrix; suppresses secondary hyperparathyroidism | Vitamin D, BMD, Fractures, Adjuvant Therapy | Muscaritoli et al. (2021); Motlaghzadeh & Wu (2024) |
| **Bioactive Phytochemicals** | **Phytoestrogens & Isoflavones** *(Genistein, Daidzein, Soy)* | 14 | 1.82% | Selective ERβ agonist activity; downregulates RANKL/OPG ratio; suppresses osteoclast differentiation | Estrogen, Bone Loss, Tamoxifen, Breast Neoplasms | Barańska et al. (2022); McCann et al. (2014) |
|  | **Polyphenols & Flavonoids** *(Resveratrol, Curcumin, EGCG)* | 9 | 1.17% | Mitigates oxidative stress; inhibits NF-κB and MAPK inflammatory signaling; promotes osteoblastogenesis | Inflammation, Biomarkers, Quality of Life, Bone Health | de Sire et al. (2022); Picon-Ruiz et al. (2017) |
| **Microbiome & Functional Agents** | **Gut Microbiota & Probiotics** *(Microbiome, Lactobacillus, SCFAs)* | 11 | 1.43% | Modulates the gut-bone-immune axis; regulates Treg/Th17 lymphocyte balance; enhances barrier integrity via SCFAs | Microbiota, Bone Metabolism, Arthralgia, Survivorship | Jackson et al. (2018) [Nat Commun]; de Sire et al. (2022) |
| **Systemic Dietary Patterns** | **Dietary Patterns** *(Mediterranean diet, Plant-based diet)* | 8 | 1.04% | Reduces systemic low-grade chronic inflammation; improves insulin sensitivity; reduces aromatase activity | Obesity, Adherence, Quality of Life, Survivorship | Runowicz et al. (2016); Demark-Wahnefried et al. (2012) |
|  | **Omega-3 Fatty Acids** *(PUFAs, EPA, DHA, Fish oil)* | 5 | 0.65% | Attenuates pro-inflammatory eicosanoids; inhibits marrow adipogenesis; alleviates musculoskeletal symptoms | Arthralgia, Bone Loss, Inflammation, Joint Pain | Hershman et al. (2015); Hertz et al. (2022) |

Abbreviations: AIBL, aromatase inhibitor-induced/associated bone loss; BMD, bone mineral density; DHA, docosahexaenoic acid; EGCG, epigallocatechin gallate; EPA, eicosapentaenoic acid; ERβ, estrogen receptor beta; MAPK, mitogen-activated protein kinase; NF-κB, nuclear factor kappa B; OPG, osteoprotegerin; PTH, parathyroid hormone; PUFAs, polyunsaturated fatty acids; RANKL, receptor activator of nuclear factor kappa B ligand; SCFAs, short-chain fatty acids; VDR, vitamin D receptor.

Corpus Proportion: Calculated against the total finalized dataset of included studies (N=768). Frequencies reflect records containing the indicated entities within the unified author keywords (DE) and extended keyword fields after regular expression standardization and synonym merging.

Benchmark References: Bracketed numbers correspond to citations listed in the main manuscript text; unbracketed author-year citations denote pivotal studies identified within the bibliographic corpus.
